# Influence of Interventionists’ Experience on Radiation Exposure of Patients Who Underwent Prostate Artery Embolization: 4-Year Results from a Retrospective, Single-Center Study

**DOI:** 10.1007/s00270-020-02461-1

**Published:** 2020-04-27

**Authors:** Bernadette Maria Theresia Kriechenbauer, Tobias Franiel, Florian Bürckenmeyer, René Aschenbach, Ioannis Diamantis, Amer Malouhi, Beatrice Steiniger, Ulf Teichgräber

**Affiliations:** 1 Department of Radiology, Jena University Hospital, Jena, Germany; 2 Institut für Diagnostische Und Interventionelle Radiologie, Universitätsklinikum Jena, Am Klinikum 1, 07747 Jena, Germany

**Keywords:** Cone beam computed tomography, Embolization, therapeutic, Lower urinary tract symptoms, Prostatic hyperplasia, Radiation exposure

## Abstract

**Purpose:**

To assess radiation exposure in men undergoing prostate artery embolization (PAE) for the treatment for symptomatic, benign prostatic hyperplasia depending on growing experience of interventional radiologists over a 4-year period.

**Methods:**

A total of 250 consecutive patients underwent PAE at a single center. Data on radiation exposure [dose area product (DAP), effective dose (ED), entrance skin dose (ESD), and fluoroscopy time (FT)] were retrospectively evaluated. Primary outcomes of interest were patient radiation exposure in five consecutive groups of 50 patients each and Pearson correlation with the number of patients treated.

**Results:**

Median DAP, ED, and ESD during prostate artery embolization were significantly higher in the first compared to the second 50 patients (56 298 µGym^2^ vs. 24 709 µGym^2^, *p* < 0.001, 146.4 mSv vs. 64.2 mSv, *p* < 0.001, and 5.1 Gy vs. 2.4 Gy, *p* < 0.001, respectively). The following consecutive groups did not differ significantly from the respective preceding group in terms of DAP, ED, and ESD. Number of digital subtraction angiography series, FT, and procedure time decreased with increasing operator experience (Pearson’s *r* = − 0.240, *p* < 0.001, *r* = − 0.269, *p* < 0.001, and *r* = − 0.504, *p* < 0.001, respectively). Bilateral prostate artery embolization was associated with less ESD and shorter FT than unilateral embolization (median 2.5 vs. 3.5 Gy, *p* = 0.02, and 26 min vs. 42 min, *p* < 0.001, respectively).

**Conclusion:**

Exposure to radiation in men who underwent PAE decreased with growing operator experience and decreasing complexity of procedures.

## Introduction

Number of men suffering from lower urinary tract symptoms (LUTS) due to benign prostatic hyperplasia (BPH) increased over recent years [1]. Minimally invasive prostate artery embolization (PAE) has proven as effective and gentle alternative to transurethral resection of the prostate gland with low risk of complications such as erectile dysfunction or incontinence [2–6]. However, prostate arteries (PA) are small vessels with a high variability of origins [7, 8]. Thus, identification of PA origins, duplicated PAs, contralateral perfusion of the prostate gland, and anastomoses with vesical, rectal, or penile arteries is challenging and time-consuming [7–9]. Procedure time and number of digital subtraction angiography (DSA) acquisitions, however, are known to increase patient’s radiation exposure.

Wang et al. [7] identified cone beam computed tomography (CBCT) angiography as a useful complement to fluoroscopy and DSA. Three-dimensional reconstruction of pelvic arteries provides additional information to support PA catheterization and to prevent nontarget embolization. Thus, CBCT angiography may reduce the number of DSA acquisitions and contrast medium usage. Nonetheless, angiography and catheterization for PAE require a high level of expertise and considerable experience [3, 8]. Hacking et al. identified the operator as independent predictor for patients’ radiation dose [10]. However, prolonged radiation exposure during PAE has not been sufficiently reflected up to now.

This study was initiated to retrospectively evaluate patients’ radiation exposure from PAE with optional CBCT angiography depending on growing experience of interventionists over a period of 4 years.

## Methods

### Study Design and Setting

Consecutive patients who underwent PAE for the treatment for BPH between July 2014 and May 2018 were retrospectively included in the single-center, observational study. PAE as an alternative to prostatectomy was indicated in patients with LUTS due to BPH that considerably impaired quality of life and was resistant to medical therapy. PAE was conducted as described previously [8]. No patient was excluded from statistical analysis.

Angiography was performed with the Artis zeego Q system (software version VD11 C 180404, Siemens Healthcare, Forchheim, Germany) consisting of a C-arm-based rotating GIGALIX X-ray tube with a flat detector and collimator. To facilitate identification of the PA origins, duplicated PA, and anastomoses, optional CBCT angiography was used at the discretion of the interventional radiologist. Examination protocol included a 7-s rotational scan of 180° with an image acquisition of 60 frames per second and an initial source power of 90 kV and 210 mA. Voltage, tube current, and filtration (copper filter from 0 to 0.9 mm) during rotational scan were automatically adjusted to the individual patient. CBCT images were transferred to maximum-intensity projections to visualize 3D data (Leonardo workstation, Syngo XWP VD 11B, Syngo VH22c, Siemens Healthcare, Forchheim, Germany). Projected images served as road map to guide subsequent catheterization and embolization. No pre-interventional computer tomography or magnet resonance angiography was conducted. Conventional DSA acquisitions for selective imaging were taken in ipsilateral anterior oblique projection of 30°–40° and caudo-cranial projection of 10°–15°. Acquisition frame rate was set at three frames per second and fluoroscopy pulse rate at 7.5 pulses per second. Default settings could be changed by operator.

To identify all the possible origins of prostate arteries, catheter tip was positioned in the distal aorta. Contrast medium with an iodine concentration of 300 mg/ml was applied at a flow rate of 8 ml/s and a delay of 4 s (Solutrast 300; Bracco Imaging, Milan, Italy). After crossing the PA origin, a solution of biocompatible 250 μm Embozene microspheres (Boston Scientifics, Natick, MA, USA) was introduced via microcatheter into the PA until flow stopped. In the case of pronounced arterial anastomoses with arteries supplying bladder, rectum, or penis, 400 μm Embozene microspheres were used. Coil embolization of anastomoses was conducted at discretion of the operator. Where possible, PAE was carried out bilaterally. Control angiography scan was performed after embolization. PAE was performed by one of six senior interventional radiologists who had 4 to 15 years of experience in interventional angiography at the beginning of implementation of PAE procedures using the Artis zeego Q system. Patients were retrospectively divided into five consecutive groups of 50 patients each (group I to V) to compare radiation exposure, procedure time, and contrast medium usage during PAE.

### Study Outcome Measurements

Examination reports included number of exposures, total fluoroscopy time (FT), entrance skin dose, and dose area product. They were disclosed separately for 2D and 3D mode. Data were retrospectively obtained from the center’s picture archiving and communication system (PACS). Primary outcome measures of radiation exposure were dose area product (DAP [μGym^2^]), effective dose (ED [mSv]), and estimated entrance skin dose (ESD [Gy]). DAP is defined as the absorbed dose multiplied by the area irradiated. It reflects the total radiation energy transmitted to the patient. ED characterizes the stochastic cancer risk to an age- and gender-averaged reference model. To estimate ED from DAP in PAE interventions, the conversion coefficient of 0.26 mSv/Gycm^2^ based on UNSCEAR’s global survey of radiation exposure was used [11]. Reference point air kerma was referred to as ESD and used to indicate risk of skin injury [12, 13]. Reference point was located 15 cm from isocenter toward x-ray tube. Thus, entrance skin air kerma depended on gantry and table motion during the procedure. ESD neither considered backscatter nor patient’s body measurements. Secondary outcomes of interest were number of DSA acquisitions, FT, procedure time, and proportion of bilateral PAE. The FT recorded in this study referred to the 2D mode only. The procedure time was defined as the period during which the interventionist was present in the catheterization laboratory.

### Statistical Analysis

Continuous variables are reported as median and interquartile range (IQR) and were compared using Mann–Whitney U test and Kruskal–Wallis test. Pearson correlation was run to determine the strength of relationship between variables of interest and increasing experience of investigators as measured in terms of number of patients treated. Spearman correlation was used to determine the relation between individual radiologists and DAP [14]. Mixed linear regression including individual radiologists as random effect was run to assess association of selected variables with DAP. Cutoff *p* value for inclusion in the multivariable model was 0.2 followed by stepwise variable selection with an entry and removal *p* value threshold of 0.1. Multivariable regression was adjusted for individual radiologists. A two-sided value of *p* < 0.05 indicated statistical significance. Categorical variables were compared by Chi-squared test. Statistical analysis was performed using SPSS Statistics 25.0 (IBM, Armonk, NY, USA) and R (R Core Team 2019, Vienna, Austria).

## Results

A total of 250 consecutive men (median age 68.9 [IQR: 11.7] years) who underwent PAE at a single center were retrospectively enrolled. Median international prostate symptom score (IPSS) was 23 (IQR: 9), and median quality of life according to IPSS question 8 was 5 (IQR: 1). LUTS included decreased peak urinary flow (median Qmax 9.2 [IQR: 5.7] ml/s) and increased prostate volume (median 60 [IQR: 37] cm^3^), (Table 1). Body mass index (BMI) and severity of LUTS were well balanced across consecutive groups (Table 2). Additional CBCT angiography in preparation for the intervention was conducted in 202 (80.8%) men. CBCT was used less frequently in the last three groups (group I–II: 96/100 (96.0%) vs. group III–V: 106/150 (70.7%), *p* < 0.001), (Table 3). Each of the six interventionists conducted 53.2% (133), 26.8% (67), 9.2% (23), 6.4% (16), 4% (10), or 0.4% (1) of PAE procedures, respectively. Spearman correlation revealed negligible correlation between individual radiologist and DAP (*r*_*s*_ = 0.074, *p* = 0.24).

**Table 1:** Patient baseline characteristics

Age, years (*n* = 250)	68.9 (11.7)
Body mass index, kg/m^2^ (*n* = 240)	26.5 (5.3)
Peak urinary flow, ml/s (*n* = 224)	9.2 (5.7)
Micturition volume, ml (*n* = 202)	173 (127)
Time of micturition, s (*n* = 172)	44 (37)
Post-void residual, ml (*n* = 205)	60 (80)
Urinary catheter (*n* = 250)	14 (5.6)
PSA, ng/ml (*n* = 247)	2.99 (3.26)
Prostate volume^a^, cm^3^ (*n* = 246)	60 (37)
Prostate artery > 1	
Right	4/250 (1.6)
Left	6/250 (2.4)
IIEF-5 score, 1 to 25 (*n* = 220)	16 (15)
IIEF-EF score, 1 to 30 (*n* = 216)	20 (17)
IPSS score, 0 to 35 (*n* = 240)	23 (9)
QoL (IPSS question 8), 0 to 6 (*n* = 240)	5 (1)

Continuous values are presented as median (interquartile range) and categorical values as counts (percentage)

*IIEF* international index of erectile function, *IPSS* international prostate symptom score, *PSA* prostate- specific antigen, *QoL* quality of life

^a^Determined by transrectal ultrasound or magnetic resonance tomography

**Table 2:** Body mass index and lower urinary tract symptoms across consecutive patient groups

	Group I (1–50)	Group II (51–100)	Group III (101–150)	Group IV (151–200)	Group V (201–250)	*p* value
Body mass index, kg/m^2^	27.1 (5.4) *n* = 49	27.1 (6.3) *n* = 49	25.8 (3.5) *n* = 47	26.5 (3.5) *n* = 47	25.4 (5.2) *n* = 48	*p* = 0.78
Prostate volume, cm^3^	60 (57) *n* = 47	60 (39) *n* = 50	65 (30) *n* = 50	60 (40) *n* = 49	57 (40) *n* = 50	*p* = 0.61
Peak urinary flow, ml/s	8.7 (8.6) *n* = 32	9.8 (5.1) *n* = 47	9.1 (6.3) *n* = 48	9.9 (6.0) *n* = 48	9.0 (5.7) *n* = 49	*p* = 0.99
Micturition volume, ml	151 (189) *n* = 11	213 (143) *n* = 48	158 (120) *n* = 47	159 (140) *n* = 47	175 (118) *n* = 49	*p* = 0.30
Time of micturition, s	33 (27) *n* = 5	45 (39) *n* = 43	40 (37) *n* = 44	47 (43) *n* = 42	44 (34) *n* = 38	*p* = 0.74
Post-void residual, ml	50 (65) *n* = 29	59 (81) *n* = 46	70 (83) *n* = 41	60 (97) *n* = 44	80 (100) *n* = 45	*p* = 0.75

Values are presented as median (interquartile range)

**Table 3:** Radiation exposure and procedure characteristics of five consecutive patient cohorts who underwent prostate artery embolization

	Group I (1–50)	Group II (51–100)	*p* value^a^	Group III (101–150)	*p* value^b^	Group IV (151–200)	*p* value^c^	Group V (201–250)	*p* value^d^
DAP, μGym^2^	56 298 (36 852)	24 709 (34 427)	*p* < 0.001	27 937 (15 875)	*p* = 0.73	25 957 (25 036)	*p* = 0.63	20 942 (18 829)	*p* = 0.25
ED, mSv	146.4 (95.8)	64.2 (89.5)	*p* < 0.001	72.6 (41.3)	*p* = 0.73	67.5 (65.1)	*p* = 0.63	54.4 (49.0)	*p* = 0.25
ESD, Gy	5.1 (3.6)	2.4 (2.8)	*p* < 0.001	2.5 (2.2)	*p* = 0.56	2.4 (2.0)	*p* = 0.69	2.2 (2.3)	*p* = 0.45
CBCT conducted	46 (92%)	50 (100%)	*p* = 0.12	40 (80%)	*p* < 0.001	33 (66%)	*p* = 0.18	33 (66%)	*p* > 0.99
Series of DSA, no	28 (15)	24 (14)	*p* = 0.11	23 (13)	*p* = 0.44	20 (11)	*p* = 0.13	20 (8)	*p* = 0.68
DAP from 2D- mode, %^e^	82 (14)	72 (30)	*p* = 0.09	77 (20)	*p* = 0.75	80 (33)	*p* = 0.10	75 (43)	*p* = 0.40
ESD from 2D-mode, %^e^	93 (5)	90 (13)	*p* = 0.45	92 (9)	*p* = 0.62	94 (11)	*p* = 0.58	92 (17)	*p* = 0.58
Fluoroscopy time, min	38 (32)	30 (31)	*p* = 0.08	29 (19)	*p* = 0.94	25 (26)	*p* = 0.14	24 (24)	*p* = 0.93
Procedure time, min	175 (71)	128 (62)	*p* < 0.001	110 (60)	*p* = 0.08	97 (61)	*p* = 0.11	100 (46)	*p* = 0.90
Contrast medium, ml	110 (70)	120 (50)	*p* = 0.31	110 (40)	*p* = 0.14	100 (56)	*p* = 0.02	100 (30)	*p* = 0.94
Bilateral PAE	41 (82%)	42 (84%)	*p* = 0.79	36 (72%)	*p* = 0.15	39 (78%)	*p* = 0.49	43 (86%)	*p* = 0.30

Values are presented as median (interquartile range), categorical values as counts (percentage)

*CBCT* cone beam computed tomography*; DAP* dose area product; *DSA* digital subtraction angiography; *ED* effective dose; *ESD* entrance skin dose

^a^*p* values apply to group I versus group II, ^b^ to group II versus group III, ^c^ to group III versus group IV, ^d^ to group IV versus group V, ^e^ also patients without CBCT included

**Fig. 1:**
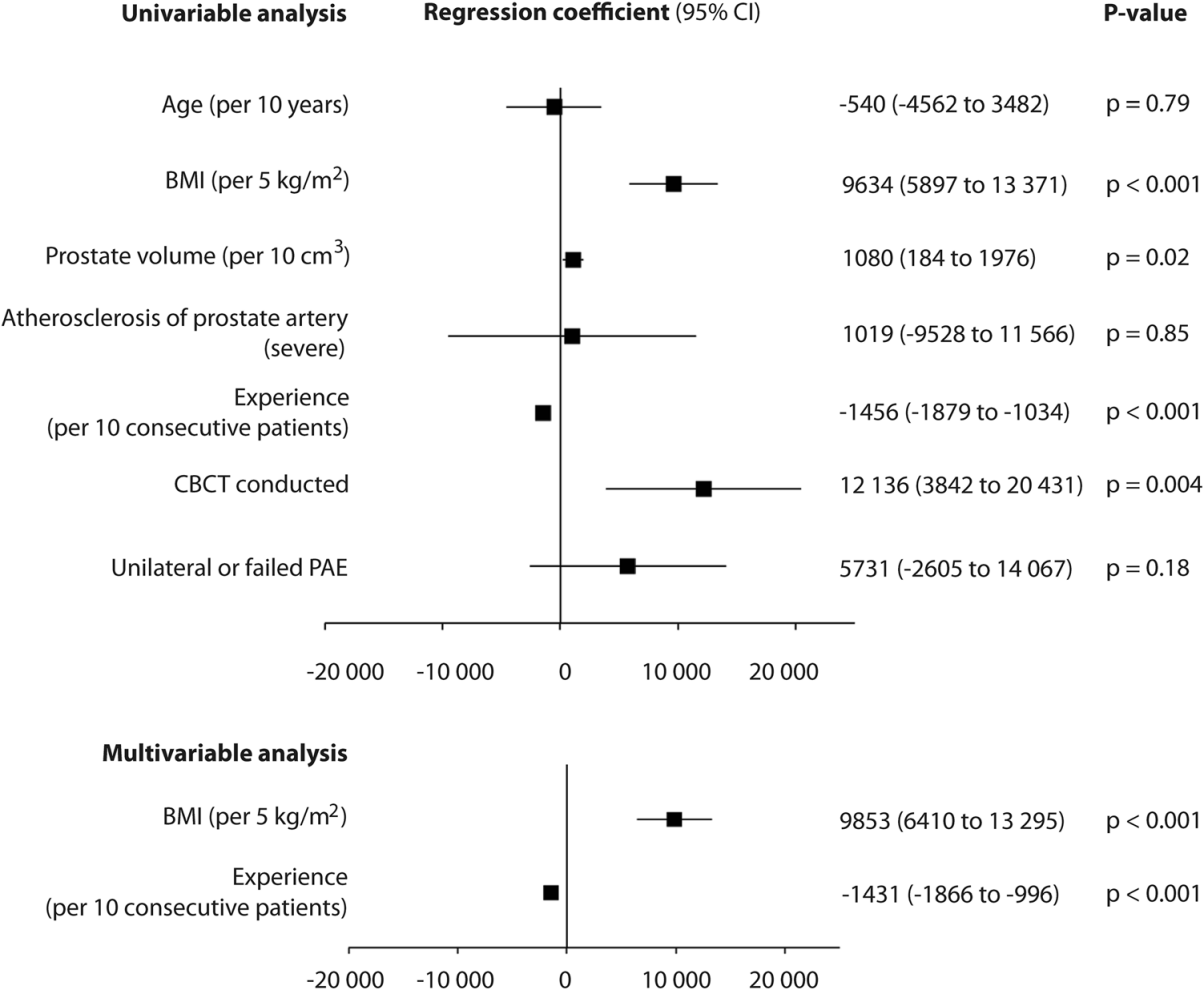
Association of DAP with selected variables evaluated by linear regression adjusted for random effect of individual radiologists. Regression coefficient represents the mean change in DAP for one unit of change in the predictive variable or for switching from one category of the predictive variable to the other. *BMI* body mass index, *CBCT* cone beam computed tomography, *DAP* dose area product, *PAE* prostate artery embolization

Median DAP was 28 612 (IQR 31 416) μGym^2^ (mean: 36 648 ± 26 610 μGym^2^). CBCT contributed to a median of 25.6% (IQR 20.0%) of DAP. Regression analysis revealed an increase of DAP by 12 136 μGym^2^ (95% CI 3842 to 20 431) in patients who underwent CBCT. Multivariable analysis showed a positive association of BMI (9853 μGym^2^ per 5 kg/m^2^, *p* < 0.001) and a negative association of investigators’ experience (− 1431 μGym^2^ per ten consecutive patients, *p* < 0.001) with DAP (Fig. 1).

**Fig. 2:**
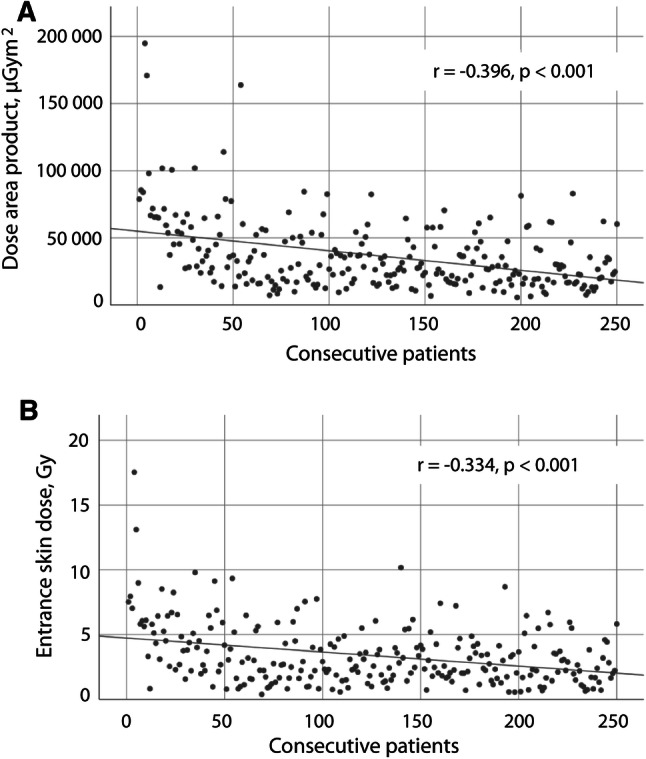
Dose area product (**A**), entrance skin dose (**B**), in consecutive patients who underwent prostate artery embolization over a 4-year period. Linear regression fits a line through data points, and Pearson correlation (*r*) describes the strength of the relationship between number of patients treated so far and the respective dependent variable

The first 50 patients (group I) were exposed to a significantly higher DAP compared to the following group of 50 patients (group II) (56 298 [IQR: 36 852] μGym^2^ and 24 709 [IQR: 34 427] μGym^2^, respectively, *p* < 0.001). Median DAP decreased with increasing experience, however, without significant differences between the following patient groups II to V (Table 3). Overall, there was a moderate correlation between DAP and the number of patients treated (Pearson’s *r* = − 0.396, *p* < 0.001), (Fig. 2A). Increasing prostate volume was weakly correlated with DAP (Pearson’s *r* = 0.150, *p* = 0.02).

Median ED was 74.4 (IQR 81.7) mSv. Group I was exposed to a significantly higher ED than group II (146.4 [IQR: 95.8] mSv vs. 64.2 [IQR: 89.5] mSv, *p* < 0.001). Afterward, there were no differences between the respective preceding groups (Table 3). Correlations with operators’ experience and prostate volume matched those of DAP.

Median ESD was 2.7 (IQR: 2.8) Gy with a CBCT contribution of 9.1% (IQR 8.8%). It was highest in the first 50 patients (5.1 [IQR: 3.6] Gy) and differed significantly from the second group (2.4 [IQR: 2.8] Gy, *p* < 0.001). The following groups did not differ significantly from the respective preceding group (Table 3). Overall, ESD decreased with operators’ experience (Pearson’s *r* = − 0.334, *p* < 0.001), (Fig. 2B) and increased with prostate volume (Pearson’s *r* = 0.125, *p* = 0.05). No radiation-induced injuries were reported.

**Fig. 3:**
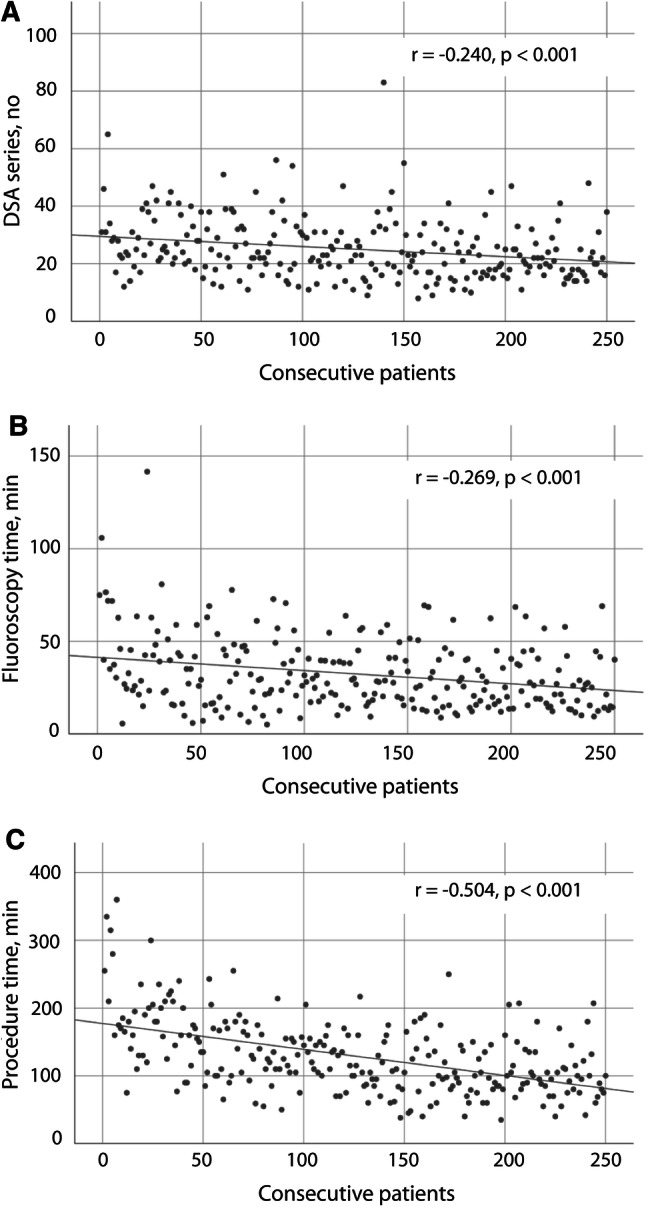
Number of DSA series (**A**), fluoroscopy time (**B**), and procedure time (**C**) in consecutive patients who underwent prostate artery embolization over a 4-year period. Linear regression fits a line through data points, and Pearson correlation (*r*) describes the strength of the relationship between number of patients treated so far and the respective dependent variable. *DSA* digital subtraction angiography

Number of DSA series and FT decreased with increasing investigators’ experience (Pearson’s *r* = − 0.240, *p* < 0.001 and Pearson’s *r* = − 0.269, *p* < 0.001, respectively), (Table 3, Fig. 3A, B). Median procedure time was 120 (IQR 70) min. It was significantly longer in the first 50 patients compared to the second group (175 [IQR: 71] min vs. 128 [IQR: 62] min, *p* < 0.001). The following groups did not differ from the respective preceding group (Table 3). Procedure time decreased along with increasing experience of the investigators (Pearson’s *r* = − 0.504, *p* < 0.001), (Fig. 3C).

The majority of patients (80.4%, 201 of 250) underwent bilateral prostate artery embolization. Unilateral embolization was conducted in 47 patients (due to stenosis at the origin of one prostate artery in 31 patients, due to dissection in three patients, due to vasospasm in one patient, and for unknown reason in 12 patients). Embolization failed completely in two patients (seventh and 111th patient). Unilateral compared to bilateral embolization entailed higher radiation exposure, increased number of DSA series, longer fluoroscopy and procedure time, and increased use of contrast medium (Table 4).

**Table 4:** Radiation exposure and procedure characteristics depending on accomplished embolization

	Total cohort	Bilateral embolization	Unilateral embolization	*p* value uni- versus bilateral	Failed embolization
Procedures	250 (100%)	201 (80.4%)	47 (18.8%)		2 (0.8%)
DAP, μGym^2^	28 612 (31 416)	27 636 (30 199)	34 786 (36 656)	*p* = 0.12	52,093
ED, mSv	74.4 (81.7)	71.9 (78.5)	90.4 (95.3)	*p* = 0.12	135.4
ESD, Gy	2.7 (2.8)	2.5 (2.6)	3.5 (3.2)	*p* = 0.02	5.4
Fluoroscopy time, min	28 (25)	26 (24)	42 (29)	*p* < 0.001	50
Series of DSA, no	23 (3)	22 (12)	28 (18)	*p* = 0.001	30
Procedure time, min	120 (70)	110 (74)	146 (60)	*p* < 0.001	260
Contrast medium, ml	111 (40)	105 (40)	120 (60)	*p* = 0.01	133

Continuous values are presented as median (interquartile range) and categorical values as counts (percentage)

*DAP* dose area product, *DSA* digital subtraction angiography, *ED* effective dose, *ESD* entrance skin dose

## Discussion

This study shows that radiation exposure during PAE was significantly highest in the first 50 patients treated after implementation of PAE using an advanced system for interventional imaging with optional CBCT function. DAP, ED, and procedure time were moderately, and ESD, number of DSA series, and FT weakly negatively correlated with increasing experience of the operating team. Technical failure of PAE on one or both sides was related to increased radiation exposure.

Median DAP in the first 50 patients slightly exceeded previously reported DAP from PAE procedures (13,440 to 45,070 μGym^2^) [15, 16]. From then on, DAP decreased to about half of the value. Our study confirmed the well-established fact that obese patients require higher doses for adequate imaging [17]. Effective dose calculation in this study accounted for stochastic risk from radiation exposure. However, it does not predict cancer risk because it does not apply to individual subjects including specific parameters that affect sensitivity to radiation such as autoimmune disease, diabetes mellitus, or hyperthyroidism [17]. Weighting factors are averaged across all ages and both genders of a standard population [18]. Patients in their seventh decade of life are supposed to have about one-fifth of risk of cancer compared to the general population [19].

In this study, radiation exposure from CBCT angiography was somewhat higher compared to previous findings. Schott et al. determined a DAP of 4070 μGym^2^ from CBCT that results in an ED of 11.8 mSv (about 30% of the total procedural irradiation), Wang et al. reported on ED of up to 24 mSv per CBCT acquisition [7, 15], and Desai et al. retrospectively assessed an ED of 14.6 mSv per PAE procedure [20]. ED from CBCT was found to be significantly lower compared to conventional CT angiography for PAE preparation (19.3 mSv, *p* < 0.01) [20]. However, ED from CBCT should be kept in mind [17]. Although CBCT considerably contributed to patient’s radiation exposure, DAP and ESD decreased significantly from group I to II though all group II patients underwent CBCT. Findings from CBCT and acquired skills and capabilities of interventional radiologists reduced the need for DSA acquisitions that cause the largest share of procedure irradiation [15–17]. Moreover, CBCT may have prevented nontarget embolization accompanied with considerable morbidity and may have increased the procedural success due to identification of duplicated PAs.

Previous studies reported on a mean peak skin dose of 2.1 to 2.6 Gy [10, 16, 21]. In contrast, ESD is a cumulative measure and expected to overestimate the peak skin dose, particularly, in the case of substantial gantry motion. However, median ESD in our study nearly reached the first notification level of 3 Gy described by Balter and Miller [22]. In patients who underwent unilateral PAE, first notification level of ESD was exceeded. The substantial radiation dose level of 5 Gy that marks the threshold to risk of clinically important skin injury was exceeded in the first 50 patients. Additionally, it should be taken into account that ESD, in contrast to PSD, does not consider backscatter [23]. Thus, risk of skin reaction from radiation exposure during PAE should not be underestimated. Our study revealed a share of 9% of total ESD from CBCT. From previous patient and anthropomorphic phantom measures on CBCT, PSD of up to 7 cGy was assumed for standard imaging protocols and thus, considered negligible. This probably is due to the distribution of radiation over 180° of the body [7].

FT in previous studies was 31 to 41 min [10, 16]. This was broadly in line with findings of our study, however, with a substantially lower FT in the last 100 patients reflecting growing experience of the operating team. Procedure time of the first 50 patients exceeded data from earlier studies (84 to 145 min) [10, 15, 21].

This study was a single-center experience. Learning curve depends on expertise and professional experience of investigators as well as on technical equipment of catheterization laboratories and thus may differ across centers. Dose parameters were not obtained from dosimeters but from the imaging system. Therefore, they should be considered as estimates. Due to the retrospective study design, only few data were available on anatomic complexity including PA origin, duplicated PA, or contralateral perfusion, on internal iliac artery atherosclerosis, on the number of embolized arteries, and on nontarget embolization. These variables might have impacted radiation exposure [10, 24].

Growing experience of investigators in performing PAE using an advanced interventional imaging system including optional CBCT angiography was associated with decreased procedure time and radiation exposure particularly after the first 50 patients treated. Unilateral embolization and increased BMI were related to higher radiation exposure.
